# Relevance of Phytochemical Taste for Anti-Cancer Activity: A Statistical Inquiry

**DOI:** 10.3390/ijms242216227

**Published:** 2023-11-12

**Authors:** Teodora-Cristiana Grădinaru, Marilena Gilca, Adelina Vlad, Dorin Dragoș

**Affiliations:** 1 Department of Functional Sciences I/Biochemistry, Faculty of Medicine, Carol Davila University of Medicine and Pharmacy, 050474 Bucharest, Romania; teodora.gradinaru@drd.umfcd.ro; 2 Department of Functional Sciences I/Physiology, Faculty of Medicine, Carol Davila University of Medicine and Pharmacy, 050474 Bucharest, Romania; adelina.vlad@umfcd.ro; 3 Department of Medical Semiology, Faculty of Medicine, Carol Davila University of Medicine and Pharmacy, 020021 Bucharest, Romania; dorin.dragos@umfcd.ro; 4 1st Internal Medicine Clinic, University Emergency Hospital Bucharest, Carol Davila University of Medicine and Pharmacy, 050098 Bucharest, Romania

**Keywords:** taste, taste receptors, bitter, sweet, cancer, anti-inflammatory, phytochemical

## Abstract

Targeting inflammation and the pathways linking inflammation with cancer is an innovative therapeutic strategy. Tastants are potential candidates for this approach, since taste receptors display various biological functions, including anti-inflammatory activity (AIA). The present study aims to explore the power different tastes have to predict a phytochemical’s anti-cancer properties. It also investigates whether anti-inflammatory phytocompounds also have anti-cancer effects, and whether there are tastes that can better predict a phytochemical’s bivalent biological activity. Data from the PlantMolecularTasteDB, containing a total of 1527 phytochemicals, were used. Out of these, only 624 phytocompounds met the inclusion criterion of having 40 hits in a PubMed search, using the name of the phytochemical as the keyword. Among them, 461 phytochemicals were found to possess anti-cancer activity (ACA). The AIA and ACA of phytochemicals were strongly correlated, irrespective of taste/orosensation or chemical class. Bitter taste was positively correlated with ACA, while sweet taste was negatively correlated. Among chemical classes, only flavonoids (which are most frequently bitter) had a positive association with both AIA and ACA, a finding confirming that taste has predictive primacy over chemical class. Therefore, bitter taste receptor agonists and sweet taste receptor antagonists may have a beneficial effect in slowing down the progression of inflammation to cancer.

## 1. Introduction

Taste receptors (TASRs) are sensory molecular structures specialized for detecting the five basic tastes: sweet, sour, salty, bitter, and umami. Receptors for sweet, umami, and bitter tastes belong to the G protein-coupled receptor superfamily, while salty and sour taste receptors are ion channels [1,2]. Although certain traditional medical systems (e.g., Ayurveda, Traditional Chinese Medicine) consider pungency and astringency to be fundamental tastes, modern science does not recognize them as basic taste sensations. Instead, they are classified as trigeminal orosensations that belong to chemesthesis, which is defined as a general sensitivity of the mucosal surfaces or skin [3]. Certain members of the transient receptor potential (TRP) channels family are the main transducers of chemestetic sensations [4]. A growing body of evidence shows that TASRs, as well as other orosensation transducers (e.g., TRPs), have widespread extraoral expressions (e.g., liver, pancreas, stomach, heart, brain, respiratory system, kidney, urinary bladder, adipose tissue, thyroid, gonads, spermatozoa, lymphocytes) and play important non-gustative, tissue-specific biological roles, such as the regulation of gastrointestinal functions, immunity, endocrine secretions, muscle relaxation, etc. [3,5,6,7]. Tastants binding to TASRs activate downstream signaling pathways in the target cells, leading eventually to either a gustatory chemosensation or to various extra-gustatory effects. They may be exogenous (e.g., nutrients, phytochemicals, xenobiotics) or endogenous (e.g., bile acids) compounds [8], and are characterized by an extreme structural and functional heterogeneity [9]. The functional diversity of TASR agonists led to the hypothesis that tastants have pharmacological significance and may even, in the future, become drug-like effectors in the treatment of various diseases [10,11,12].

Several studies have shown a direct involvement of bitter taste receptors in inflammatory processes. For instance, TAS2R16 expression is elevated in inflammatory conditions, and salicin, a TAS2R16 agonist, antagonizes NF-kB signaling [13]. Tiroch et al. demonstrated that resveratrol exhibits its anti-inflammatory activities (IL-6 targeting pathway) through the activation of TAS2R50 [14].

There is also increasing evidence about the multifaceted involvement of bitter taste receptors in cancer [15,16]. Recent studies have shown that bitter taste receptors are expressed differently in cancerous tissues compared to normal, cancer-free tissues [17,18,19,20]. In most cases, the expression of these receptors is downregulated. Additionally, overexpression of TAS2R has been shown to have anti-cancer effects [19].

Cancer represents a massive healthcare burden worldwide [21]. Intense research efforts have been focused on finding new methods of intervention in cancer prevention and treatment, in the quest for new therapeutic molecular targets that can modulate mechanisms involved in carcinogenesis. Targeting various pathways linking inflammation with cancer represents such an innovative therapeutic strategy.

In relation to cancer, inflammation can be either beneficial or detrimental to the body. Acute inflammation has been shown to inhibit tumor growth and is effective in treating various types of cancers, including bladder and colorectal cancers [22,23]. However, chronic inflammation can predispose the body towards cancer development. Chronic inflammation sustains and stimulates every step of carcinogenesis by promoting mutagenesis, tumor immune escape, inactivation of tumor suppressors, cell proliferation, etc., whereas cancer initiates, facilitates, and maintains local inflammatory processes through the chemoattraction of inflammatory cells, damage-associated molecular patterns, and hypoxia, which favors tumor growth and dissemination [24,25,26]. For instance, chronic gastritis increases the risk of stomach cancer [26,27,28]. Scientists estimate that at least 20–25% of cancers are produced or influenced by chronic inflammation [29,30], which may be linked to chronic infections with various viruses [31,32,33,34], Helicobacter pylori [35], autoimmune diseases [36], metabolic disorders [37], or exposure to various chemicals [38,39]. Tumor-promoting inflammation and genomic instability were considered to be the first two main “enabling characteristics” or oncogenic drivers which allow evolving preneoplastic cells to develop and acquire the aberrant phenotypic traits in the course of tumor growth and progression. Since then, other “enabling characteristics” have emerged (e.g., non-mutational epigenetic reprogramming, polymorphic microbiomes), adding more complexity to the cancer pathogenesis [40,41].

Several molecular targets, including transcription factors (e.g., NF-kB, STAT-3, AP-1), enzymes (e.g., COX-2, MAPK), and receptors (e.g., E series of prostaglandin receptors—EP receptors) were found to be simultaneously involved in inflammation and carcinogenesis, as well as in the progression of chronic inflammation towards cancer [25,42,43,44,45,46].

Plants are a huge reservoir of health-promoting compounds. At least 60% of approved anti-cancer drugs are derived from medicinal plants [47]. Bitter phytochemicals have displayed anti-cancer activity (ACA) in many in vitro and in vivo studies, with some of them showing the direct contribution of TAS2Rs activation to this effect [18,19,20]. In several cases (e.g., resveratrol, curcumin, kaempferol, epigallocatechin gallate) this ACA of bitter phytocompounds was proven to be achieved by also acting on inflammatory signaling pathways [48,49,50,51,52]. Regarding the anti-inflammatory potential of phytochemicals, our group has previously shown that bitter phytocompounds have a higher probability of displaying anti-inflammatory activity (AIA), when compared to non-bitter phytocompounds, while a sweet taste is negatively corelated with this activity [53]. In another study, we reported that the taste of phytocompounds is a better predictor of the ethnopharmacological activities of the medicinal plants than the phytochemical class [54].

By using data mining and statistical tools, the current research aims to determine whether there is any concordance between the taste or chemical class of phytocompounds and their evidence-based ACA, as well as between evidence-based ACA and AIA of phytocompounds. If there is a positive AIA-ACA agreement, the study will investigate whether taste or chemical class can predict this. Finally, the study aims to determine which attribute of phytocompounds (either chemical class or taste) better predicts a potential AIA-ACA agreement.

## 2. Results

Our analysis relies on the information gathered and made public in the PlantMolecularTasteDB (PMTDB) (http://plantmoleculartastedb.org, accessed on 1 February 2023), a previously published work, a database which contains a total of 1527 phytocompounds [9]. PMTDB is the largest database dedicated to individual plant-derived tastants (referred to as “phytotastants” in this paper) and orosensation-active phytochemicals among all the databases focused on flavor chemistry. In contrast, most databases are mixed collections of tastants (natural and synthetic, phytocompounds and synthetic drugs) or odorants [55], despite being sometimes larger (e.g., ChemTastesDB) [56].

The total number of phytocompounds included in the statistical analysis was 624: 429 with proven AIA and ACA, 82 with proven AIA but no ACA, 32 with proven ACA but not AIA, and 81 without either AIA or ACA. The total number of phytocompounds that fulfilled the inclusion criterion (a minimum of 40 hits in a PubMed search using the name of the phytotastant as the keyword, for details see Section 4, Materials and Methods) was obviously higher than that found in our previous study, mentioned in the Introduction section [53]. Hydroxy-α-sanshool, caffeic acid ethyl ester, kaempferol-3-O-β-galactopyranoside, guaiaverin, and clovamide are a few examples of such newly introduced phytotastants in our statistical analysis.

Regarding the update of data on AIA recorded in the PMTDB, we took notice of new studies about the AIA of various compounds that were available after the PMTDB was publicly open (e.g., isoschaftoside [57], cis-aconitic acid [58]).

### 2.1. The Correlation between Anti-Inflammatory/Anti-Cancer Activity and Taste/Chemical Class

Among the six tastes/orosensations included in our study (bitter, sweet, sour, salty, pungent, astringent), only bitter taste had a statistically significant positive association with both AIA and ACA. This was reflected by the greater-than-one odds ratio (OR) with a 95% confidence interval (CI) not including one. Conversely, sweet taste had a statistically significant negative association with ACA, as reflected by the less-than-one OR with a 95% confidence interval not including one. Sweet taste might have a negative association with AIA, but the *p*-value for this latter association was higher than the threshold calculated according to Bonferroni correction (Table 1).

Among the chemical classes, only flavonoids have a statistically significant positive association with both AIA and ACA (OR > 1.95% CI 3.03–324.24). By contrast, the saccharides and alkaloids have a negative association with AIA, and saccharides and amino acids have a negative association with ACA, as reflected by the less-than-one OR with a 95% CI not including one (Table 2).

The analysis was limited to flavonoids to determine whether the positive association between flavonoids and AIA was mediated by a specific taste, and the result was statistically significant for bitter taste only (see the row “bitter (flavonoids only)” in Table 1). A similar computation was done for alkaloids, and no association was found between the pharmacodynamic activity and taste. The same holds for amino acids.

For saccharides, sweet taste might be conceived as the mediator of the association with the absence of AIA/ACA; however, this cannot be proven statistically, as all but one saccharide (gentiobiose) are sweet.

Pooling together chemical classes did not influence these results (Table 3).

### 2.2. Correlation between Anti-Cancer and Anti-Inflammatory Activity

The statistical analysis revealed a strong correlation between ACA and AIA, which was maintained over the entire range of tastes/orosensations (Table 4) and chemical classes (Table 5), with the exception of amino acids.

In Table 4, all results are either extremely high (for All Tastes and for Bitter) or highly statistically significant. AIA-ACA association holds, irrespective of taste/orosensation or chemical class.

As illustrated in Table 5, the odds ratio (OR) is equal to zero for the chemical classes in which no phytocompound was devoid of both AIA and ACA (a = 0) (coumarins, diterpenoids, monoterpenoid glycosides, triterpenoid glycosides). For all these chemical classes, the vast majority of phytocompounds have both AIA and ACA (d is disproportionately higher than a, b, and c). For all the other chemical classes, OR is greater than one (and sometimes even infinite), but it does not reach the level of statistical significance due to the (very) low count of phytocompounds devoid of both AIA and ACA. The *p*-value is higher than the Bonferroni corrected threshold and the less-than-one inferior limit of the 95% confidence interval for OR.

**Figure 1:**
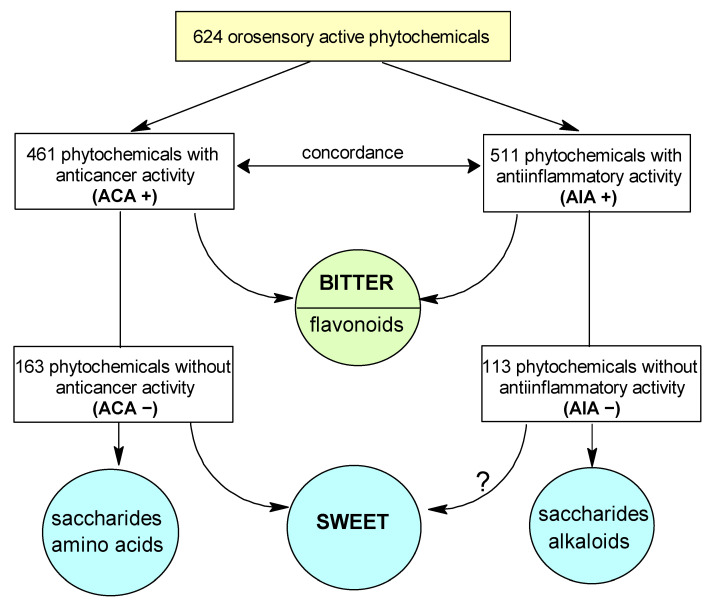
Flowchart showing the main statistical concordances found in the present study (Legend: ACA = anti-cancer activity, AIA = anti-inflammatory activity, (+) = present, (−) = absent): ACA(+) with AIA(+), bitter with ACA(+), bitter with AIA(+), flavonoids with ACA (+), flavonoids with AIA(+), sweet with ACA(−), saccharides with ACA(−), saccharides with AIA(−), amino acids with ACA(−), alkaloids with AIA(−). The association of sweet with AIA(+) is marginally statistically significant - this is the reason a question mark accompanies the correspondent curved arrow.

The most significant results are synthesized in Figure 1.

## 3. Discussion

From the 624 total phytotastants that met our inclusion criteria, ACA activity was present in 461 phytocompounds.

### 3.1. Taste as an Important Determinant of Pharmacodynamic Activity

The most relevant finding of the present study is that only bitter taste has a statistically significant positive association with both ACA and AIA. The last concordance (bitter -AIA) has already been proven in a previous study [53], performed on a smaller number of phytocompounds. The present investigation included a larger number of phytocompounds in the PMTDB because more of them met the inclusion criterion of 40 hits in PubMed. Additionally, we updated the data on the AIA of phytotastants available in the PMTDB. In line with our observation/results, there is an increasing body of evidence that supports the involvement of TAS2R agonists in alleviating the inflammatory processes [59,60] and their role as chemopreventive and/or chemotherapeutic agents [19,61]. Regarding ACA, it is relevant that the activation of various TAS2Rs subtypes (e.g., TAS2R4, TAS2R8, TAS2R13, TAS2R14, TAS2R10, and TAS2R30/47) by bitter compounds such as quinine, apigenin, noscapine, caffeine, and denatonium led to significant ACA in several cancer cell lines by multiple mechanisms: reduced cell proliferation, metabolic activity, migration, invasion, motility, metastasis and angiogenesis, increased apoptosis, and enhanced chemosensitivity to conventional anti-cancer drugs [18,19,20,61]. TAS2Rs also play a role in the tumor-restraining function of the fibroblasts found in the tumoral microenvironment. For instance, TAS2R9 is upregulated in cancer-associated fibroblasts, the most representative cellular type in the stroma of pancreatic ductal adenocarcinoma, and it was proposed as a novel molecular target, a candidate for the inhibition of cancer progression, by reprogramming the crosstalk between fibroblasts and cancer epithelial cells [62].

A secondary finding is that there was only one other relevant association that was identified: sweet taste is negatively correlated with both AIA and ACA. This association does not contradict the idea that taste is an important determinant of pharmacodynamic activity, which may be more important than the chemical class [54]. It is in fact the most important general idea suggested by us in our present study, as well as in our previous studies, regarding the relationship between tastes and (ethno)pharmacological activities [3,9,53,63].

### 3.2. Upgrade of the Previously Reported Taste—AIA Associations

Our previous study reported a negative correlation between sour taste and AIA [53], but this finding was not confirmed in the present analysis. There are several possible explanations for this discrepancy. Firstly, new studies have been published regarding the AIA of some phytochemicals, such as caproic acid [64] and cis-aconitic acid [58]. Secondly, there were different numbers of compounds that met the inclusion criteria of at least 40 hits in PubMed, such as hydroxy-α-sanshool.

### 3.3. The Pharmacodynamic Activity of Phytochemicals: Taste Has Predictive Primacy over Chemical Classes

Among the chemical classes, only flavonoids have a statistically significant positive association with both AIA and ACA. In contrast, saccharides and alkaloids have a negative association with AIA, while saccharides and amino acids have a negative association with ACA. The positive association between flavonoids and AIA/ACA does not contradict the primacy of bitter taste over chemical classes as the determinant of AIA/ACA, since the vast majority of flavonoids are bitter. Similarly, the negative association between saccharides and AIA/ACA can be explained by the fact that most saccharides are sweet.

### 3.4. Bitter—Sweet: Are They in Opposition?

Both bitter and sweet taste receptors are G-coupled protein receptors. Bitter taste receptors (TAS2R) consist of only one type of receptor and belong to the A class type receptors, while sweet taste receptors (TAS1R) are dimers formed by two different G-coupled protein receptors (TAS1R2 and TAS1R3) and belong to the C class type receptors [2,65,66].

Regarding the present contrasting results between bitter and sweet associations with AIA and ACA (positive and negative associations, respectively) reported by our study, it is interesting to note that these two tastes are frequently framed in various types of opposition, from both traditional [3] and modern medical points of view [67].

For instance, in Ayurveda, bitter taste is considered to have dry and light (decreasing weight) qualities, while sweet taste is considered to have wet/oily (emollient/moisturizing) and heavy (increasing weight) qualities [3].

Modern science points to various types of bitter–sweet opposition. Structurally, the majority of bitter tastants have a smaller size than sweet compounds, as well as higher hydrophobicity [68]. Functionally, bitter taste receptor activation can produce a biological effect, such as the secretion of antimicrobial peptides in sinonasal epithelial cells that co-express both types of taste receptors. However, this effect may be suppressed by sweet taste receptor activation [69,70,71]. Interestingly, two drugs that have similar, well-known AIA, ibuprofen and flufenamic acid, showed contrasting results regarding the affinity for TASRs: flufenamic acid was revealed as an agonist of bitter taste receptors TAS2R14 [72], while ibuprofen was a potent inhibitor of sweet taste receptors TAS1R2/TAS1R3 [73].

Also, bitter tastants seem to have many common off-targets (e.g., cytochrome P450 enzymes, carbonic anhydrases, adenosine A3 receptor, hERG potassium channel), while sweet compounds have very few [67]. In regard to caloric value, sweet foods such as fruits and vegetables have a high nutritional value, while bitter have none, or are even anti-nutritional [74]. Bitter foods such as leafy greens and cruciferous vegetables are highly nutritious, but bitter compounds in some plants can be toxic in large amounts [75]. In terms of taste preferences, bitter is innately aversive and rapidly rejected, whereas sweet is appetitive and avidly ingested [76]. Nevertheless, the inclusion of bitter items in diet was associated with health-promoting effects [77,78], while a high-sugar diet was outlined as contributing to the pathogenesis of several diseases (e.g., obesity, metabolic syndrome, and inflammatory diseases) [79].

### 3.5. Anti-Inflammatory Activity- Anti-Cancer Activity Association Is Independent of Taste

Given the very strong association between AIA and ACA, any factor associated with AIA (such as bitter taste) is expected to also be associated with ACA; of course, the reciprocal is also true. Table 4 suggests that AIA-ACA association is independent of taste as it holds over the entire spectrum of tastes/orosensations. The strong (and taste-independent) AIA-ACA association is probably due to the many molecular pathways that inflammation and neoplasia share [25,43,44,45,46]. The perpetuation of regenerative processes induced by chronic inflammation frequently degenerates in cancer [29,30].

The potential role of anti-inflammatory plant-derived agents in cancer therapy has been already hypothesized by other scientists [80]. The AIA-ACA concordance was also highlighted by a previous mixed study based on field data obtained from Indian tribal healers, chemoinformatic prediction tools (PASS database, admetSAR, CLC-pred), and in vitro experiments, which concluded that the ethnopharmacological anti-inflammatory plants may have anti-cancer potential [81]. Another research showed that synthesized quinoline glycoconjugate derivatives exhibited a positive correlation between ACA and AIA [82]. One of the most anti-inflammatory compounds against COX-2, compound C8, also had the strongest cytotoxic activity against HeLa cells [82].

Our results are also in line with data proving the central role of NF-kB and COX-2/PGE2 pathways in both inflammation and cancer. NF-kB is induced by pro-inflammatory mediators, such as TNF, IL-1, and itself controls pro-inflammatory factors gene expression, such as TNF, IL-1, IL-6, COX-2 [83]. Also, NF-kB occupies a crucial role in tumor initiation and in cancer progression and spread [84], being simultaneously a facilitator of inflammation progression towards cancer [85]. Various natural inhibitors of NF-kB signaling (e.g., polyphenols, terpenoids) displayed simultaneous anti-inflammatory and anti-cancer potential [86,87]. Therefore, some of these NF-kB inhibitors, administered as monotherapy or combined therapy, were either investigated or are under current evaluation in clinical trials for both types of conditions (e.g., the effects of curcumin in rheumatoid arthritis [88], chemotherapy- or radiotherapy-induced oral mucositis [89], and oral leukoplakia [90], as well as prostate cancer [91], metastatic tumors [92], resveratrol effects in smoking-induced inflammation [93], and multiple myeloma [94]).

Interestingly, numerous studies have provided evidence that nonsteroidal anti-inflammatory drugs (NSAIDs), including aspirin, may hold promise in helping to prevent cancer. Experimental and epidemiologic studies, along with randomized clinical trials, have shown that NSAIDs may have a prophylactic effect against certain cancers [95,96,97,98,99,100,101,102,103,104]. Also, specific anti-inflammatory therapy with canakinumab, an interleukin-1β inhibitor, significantly reduced the incidence of lung cancer in patients with atherosclerosis [105]. Few studies reported opposite effects of excessive use of certain NSAIDs [106,107].

NSAIDs are known for their COX inhibitory activity [108]. COX-2 is the inducible isoform and is linked to inflammatory processes [109], as well as to cancer [110]. Overexpression of this enzyme identified in various types of cancer, including breast, colon, and prostate cancer, was associated to poor outcomes, poor prognosis, and reduced survival rates [111,112].

The effects of COX-2 on cancer progression are often mediated via the COX-2/PGE2 pathway [25]. PGE2 is a proinflammatory factor, proven to be upregulated in cancer cells [113,114,115] that bind to EP1, EP2, EP3, and EP4 receptors. Consequently, certain downstream signaling pathways involved in tumor growth are activated, such as the PKA, β-catenin, NF-kB, or PI3K/AKT pathways [25]. Inhibition of the PGE2 pathway by targeting EP receptors is currently being evaluated as a new therapeutic strategy for cancer treatment [116,117,118].

### 3.6. Anti-Cancer Activity—Is Bitter Better?

The association of bitter taste with ACA is not surprising, since there is an important body of evidence showing that TAS2R agonists are able to exert ACA, and TAS2Rs might play an important role in carcinogenesis. In the majority of cases, TAS2R activation was associated with antitumor activity [16,18,20,61]. The anti-cancer effects of bitter taste receptor activation include the impact on apoptosis, proliferation, migration, invasion, viability, cycle cell arrest, and stemness of cancer cells, and the influence on tumor growth. There is a difference in TAS2R expression in neoplastic cells compared to normal cells. For example, quinine (through the activation of TAS2R4) and apigenin (through the activation of TAS2R14) significantly attenuated metastatic breast cancer cells proliferation and increased the number of cells in the early apoptotic states, whereas, in a normal breast epithelial cell line, these effects were absent [20]. As well, noscapine stimulation of TAS2R14 increased ovarian cancer cells apoptosis [18], while Carey et al. highlighted TAS2R’s involvement in the apoptotic process of head and neck squamous cell carcinoma cell lines [119].

To date, numerous studies show certain types of bitter taste receptors tend to be downregulated in cancer cells compared to cancer-free cell lines (TAS2R1, TAS2R4, TAS2R8, TAS2R10, TAS2R14, TAS2R38) [17,18,19,20]. Carey et al. examined the variation in expression of bitter, sweet, and umami receptors in 45 solid tumors. They reported that TAS2R4, TAS2R5, TAS2R14, TAS2R19, TAS2R20, and TAS2R31 expression was at a lower level in neoplastic tissues compared to cancer-free tissues [120]. However, some studies indicate that there is an overexpression in some bitter taste receptors in tumors. For instance, TAS2R14, TAS2R20, and TAS2R30/31/43/45/46 have been shown to be expressed more in highly metastatic cancer cell lines (MDA-MB-231) than in normal breast cell lines (MCF-10A) [121]. Another study demonstrated that, despite the tendency for some bitter taste receptors to be downregulated in cancer tissues, TAS2R38 was often overexpressed in solid tumors, as compared to normal tissues [120].

### 3.7. Anti-Cancer Activity—Is Sweet Worse?

Sweet taste is negatively correlated with ACA. Notably, a higher dietary intake of sugar, which is more rapidly absorbed than other sources of carbohydrates, may increase plasma glucose and insulin levels, which are known as risk factors for carcinogenesis [122,123]. According to a systematic review and meta-analysis of observational studies, there is a statistically significant positive correlation between the incidence of breast and prostate cancer and higher intake of sugar-sweetened beverages. The study also found a positive correlation between fruit juice consumption and the risk of prostate cancer [124]. Obesity is another redoubtable risk factor for cancer [125], while the ingestion of concentrated sugars is probably the principal cause of obesity [126]. Moreover, there is mounting evidence that caloric restriction and fasting, mimicking a low-carbohydrate, low-protein diet, are beneficial in cancer prevention by reshaping metabolism and anti-cancer immunity [127,128]. Our findings may also be consistent with the results of a recent clinical trial that found an association between increased cancer risk (all cancers, prostate cancer, breast cancer, and obesity-related cancers) and the intake of artificial sweeteners, mainly aspartame and acesulfame-K [129].

### 3.8. Limitations of the Study

Our research has several limitations that need to be considered. Firstly, the lack of studies concerning ACA may still be caused by the absence of evidence, despite our 40 hits inclusion criteria. Secondly, studies regarding ACA/AIA were excluded from this investigation if a metabolite of a specific phytocompound showed ACA/AIA activities, but the corresponding parent phytochemical did not show such activities. For instance, gluconasturtiin itself, found in cruciferous vegetables, has no evidence-based ACA and was therefore categorized in our statistical analysis as ACA(−), while its metabolite, phenylethyl isothiocyanate (PEITC), has ACA [130,131]. Thirdly, there are contradictory data regarding the positive or negative evidence of pharmacological activities of some phytochemicals. For example, morphine has both antineoplastic and protumor effects [132,133,134], which makes it difficult to categorize phytochemicals in one class or the other (positive evidence or negative evidence). Fourthly, the inclusion criterion of at least 40 hits in a PubMed search is justified by two reasons: (1) to use the same methodology as the one used in our previous study; (2) to lower the probability for a specific item to be identified as not having a biological role because it is not studied enough. On the other hand, there are phytocompounds that have either AIA or ACA but do not meet the inclusion criterion of at least 40 hits in PubMed. For example, santamarin displays both AIA and ACA [135,136,137,138], but it did not meet the inclusion criterion, and therefore it was not included in our statistical analysis. Lastly, time limitations are an important factor that can change outcomes. The literature search for our study started in February 2023 and finished in June 2023. For some phytocompounds, new studies may have been published after our search ended.

Finally, our study is limited by the lack of experimental validation of our results. Simultaneous testing of AIA and ACA of a battery of phytotastants belonging to various chemical classes and looking for potential AIA-ACA or taste -ACA/AIA or chemical class-ACA/AIA concordances, or screening phytochemicals agonists of TAS2Rs for cancer cell growth inhibitory capacity and looking for potential association between their half-maximal inhibitory concentration (IC50) values and bitter taste threshold concentrations may be interesting approaches for upcoming research initiatives.

## 4. Materials and Methods

The study relies on the information available from PMTDB (http://plantmoleculartastedb.org accessed at 1 February 2023), a public database previously published, which contains a total of 1527 phytotastants [9].

For any given compound, AIA and ACA are two of the most, and, implicitly, two of the first, studied biological activities, only second to antimicrobial activity, according to our previous study [53].

Both ACA and AIA were considered evidence-based if they were supported by at least one correctly conducted study, irrespective of being performed in vitro or on animal or human subjects. However, among the phytochemicals with positive evidence for AIA and ACA, only those that also fulfilled the inclusion criterion were included/used in the statistical analysis.

The ACA was searched for these compounds in three international databases: PubMed, Google Scholar, and ScienceDirect.

The systematic literature research was made by using the phrases: [specific phytochemical name] AND (cancer) or [specific phytochemical name] AND (chemopreventive) or [specific phytochemical name] AND (cytotoxic) or [specific phytochemical name] AND (antiproliferative) or [specific phytochemical name] AND (tumor) or [specific phytochemical name] AND (neoplastic) or [specific phytochemical name] AND (chemotherapy).

ACA was considered when that specific phytocompound had a direct activity on cancer cells or tumors, such as antiproliferative activity, induction of apoptosis, cytotoxic activity on cancer cells, lowering the viability of the cancer cells, or diminishing the tumor volume or growth. The effects on metastasis potential or the effects on invasion capability were not taken into account. Also, the effects on cancer vessels and the effects of increasing the outcome of potentiating some chemotherapy treatment or reversing chemoresistance were not considered. The effects of phytocompounds as chemosensitizers was also overlooked.

The eligible studies for our work were those using only single phytotastants. Studies using plant extracts, a combination of a phytocompound with a chemotherapeutic agent, or mixtures of two or more phytocompounds were excluded in the present research. Also, studies were excluded from this research if a metabolite of a specific phytocompound showed ACA, but that parent phytochemical did not show any anti-cancer effect. The exclusion of the phytochemical’s metabolite was necessary to evaluate the impact of taste category on the pharmacodynamic activity of the phytochemical, as differences in taste between the two could affect the results.

In case of AIA, we updated the data available in the PMTDB, performing again the search for the phytotastants recorded as having negative evidence in the PMTDB and the previous study, using the same keywords as before [53].

Taking into account that lack of AIA or ACA is difficult to identify by searching the literature, we considered as “negative evidence for AIA/ACA” the fulfilment of two criteria, a rule already used in our previous study [53]: (1) lack of any positive evidence related to the respective pharmacological activity (AIA or ACA); and (2) the PubMed search using the name of the phytotastant as a keyword produced at least 40 articles, meaning that the phytocompound has been the object of a sufficient research/number of studies for the respective pharmacological activity (AIA or ACA) to be identified, and that the lack of positive evidence is not caused by a deficiency of studies.

**Figure 2:**
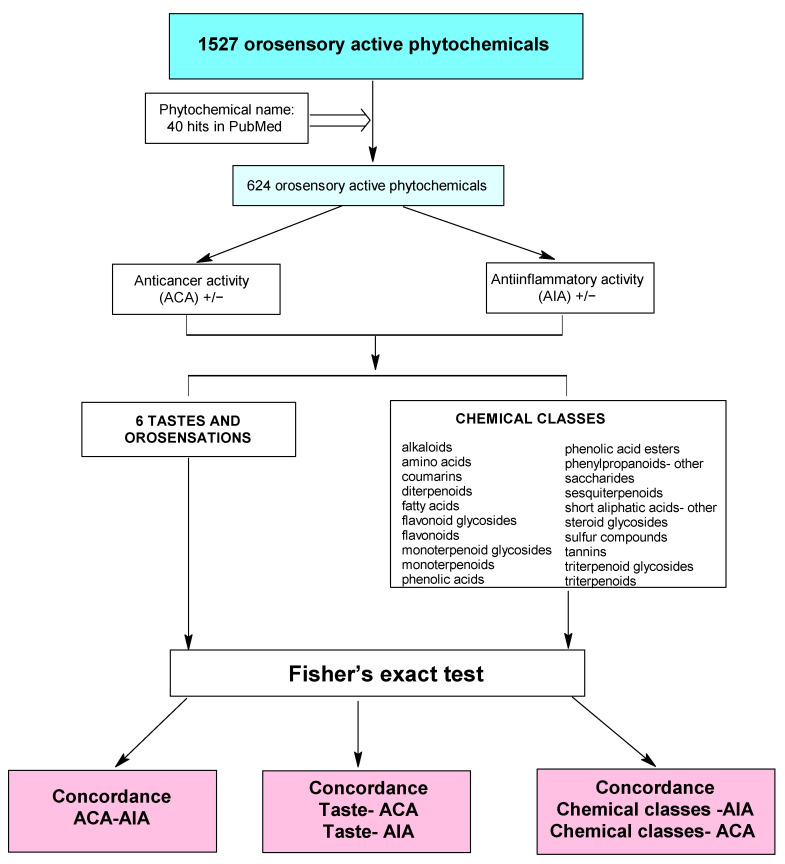
Flowchart outlining the steps of data preparation and statistical analysis (www.plantmoleculartastedb.org, accessed on 1 February 2023).

Only 624 phytotastants (either with negative or positive evidence of AIA/ACA) out of 1527 in the PMTDB fulfilled the inclusion criterion and were therefore included in the statistical analysis (Figure 2).

The correlations between two categorical parameters (such as taste and AIA/ACA or chemical class and AIA/ACA) were analyzed by means of Fisher’s exact test, performed on 2 × 2 contingency tables built on the model in Table 6. Fisher’s exact test was employed (instead of the more commonly used Chi-squared test) because, in many instances, the number of cells in these contingency tables, especially a, b, and/or c, are quite small, which result in small (<5) corresponding expected values, precluding the employment of the Chi-squared test. Moreover, Fisher’s exact test is always preferable to the Chi-squared test, as it computes the probability directly and exactly, while the Chi-squared test only approximates the probability, yielding results that are close to the true probability only when the number of cells in the contingency table are (relatively) large.

The results were considered statistically significant if the *p*-value was below the generally accepted threshold of 0.05. When multiple comparisons were performed, the significance level (commonly set at 0.05) was lowered according to Bonferroni correction: the corrected significance level was 0.05 divided by the number of comparisons. The rationale for employing Bonferroni correction is that whenever large number of comparisons are performed, there is a high probability that some results (about 1 in 20, should a threshold of 0.05 be used for *p*-value) appear significant by sheer chance, without reflecting a truth about the real world. For example, if two comparisons are performed and a threshold of 0.05 is employed, the probability that each of the comparisons yields a falsely significant result is 0.05, but the probability that at least one of the two comparisons yields such a result is 0.05 + 0.05 = 0.1, so the 0.05 threshold for *p*-value is no longer observed. However, if the threshold for the *p*-value is lowered o 0.025 (i.e., 0.0.5 divided by 2), then the probability that at least one of the two comparisons yields a falsely significant result is 0.025 + 0.025 = 0.05, so the generally accepted threshold of 0.05 is observed.

Odds ratio (OR) was used to estimate the odds that, say, a bitter phytocompound (if we look at Table 1) has, for example, ACA. An OR of 1 means that there is no relation between the bitter compound and ACA. An OR larger/smaller than 1 means that a bitter compound has a higher/lower probability to have ACA than a non-bitter phytocompound (1.92 in Table 1). In order to estimate the value in the real world, the 95% confidence interval for OR is also provided, signifying that there is a 95% probability that the value in the real world is somewhere between 1.32 and 2.77.

All the statistical calculations were performed using the R language and environment for statistical computing and graphics, version 4.0.3 (copyrighted by The R Foundation for Statistical Computing).

The chemical classes were chosen so as to be as disjunct as possible, i.e., each compound fell in one, and only one, chemical class. In other words, the phytocompounds were classified in mutually exclusive chemical classes. About two thirds of these classes (monoterpenes, lignans, organic acids, carotenoids, cyanogen glycoside, fatty aldehydes, steroids, diterpenoid glycosides, phenolic glycosides, polyketides, proteins, alkaloid glycosides, alkylamides, amides, anthraquinones, aromatic aldehydes, aromatic ketones, chromones, coumarin glycosides, fatty acid esters, fatty alcohols, high aliphatic acids, high aliphatic alcohols, lactones, medium aliphatic acids, naphthofurans, phenolic acid glycosides, sesquiterpene, vitamins, amines, anthraquinone glycosides, aromatic esters, carboxylic acids, cardenolides, cyclic polyols, guanidines, indoles, lignan glycosides, low aliphatic alcohols, monoterpene, monoterpene alcohols, organic acids esters, phenolic acid amides, phenylpropanoid glycosides, polyol phosphates, and sulfur compound glycosides) were too sparsely populated (less than 10 phytocompounds each) to be included in the statistical analysis. Those included in the statistical analysis are listed in the first column of Table 2. In order not to miss some important associations, some of these classes were then pooled together (alkaloids = alkaloids + alkaloid glycosides, anthraquinones = anthraquinones + anthraquinone glycosides, coumarins = coumarins + coumarin glycosides, cyanogen = cyanogen + cyanogen glycosides, diterpenoids = diterpenoids + diterpenoid glycosides, fatty compounds = fatty acids + fatty acid esters + fatty aldehydes + fatty alcohols + high aliphatic acids + high aliphatic alcohols, flavonoids = flavonoids + flavonoids glycosides, lignans = lignans + lignan glycosides, monoterpenoids = monoterpenoids + monoterpenoids glycosides + monoterpene + monoterpene alcohols + monoterpenes, phenolic acids = phenolic acids + phenolic acid glycosides + phenolic acids esters + phenolic acid amides, phenolic others = phenolic others + phenolic glycosides, phenylpropanoids (others) = phenylpropanoids (others) + phenylpropanoid (other) glycosides, polyols = polyols + polyol phosphates, sesquiterpenoids = sesquiterpenoids + sesquiterpene, steroids = steroids + steroids glycosides + cardenolides, sulfur compounds = sulfur compounds + sulfur compound glycosides, triterpenoids = triterpenoids + triterpenoids glycosides). Still, more than half of the resultant classes (lignans, organic acids, cyanogen, carotenoids, proteins, polyketides, anthraquinones, vitamins, naphthofurans, medium aliphatic acids, lactones, chromones, aromatic ketones, aromatic aldehydes, amides, alkylamides, polyols, phenolic acid esters, organic acids esters, low aliphatic alcohols, indoles, guanidines, cyclic polyols, carboxylic acids, aromatic esters, and amines) included less than 10 phytocompounds and were consequently eliminated from the statistical analysis.

The classes that were pooled together were: alkaloids, flavonoids, monoterpenoids, triterpenoids, saccharides, phenolic acids, amino acids, sesquiterpenoids, coumarins, phenolic others, fatty compounds, short aliphatic acids, diterpenoids, steroids, tannins, sulfur compounds, and phenylpropanoids (others).

## 5. Conclusions

The present study demonstrates a strong association between ACA and AIA in phytocompounds. In other words, an anti-inflammatory phytocompound is more likely to also have ACA. This outcome supports the idea that targeting inflammation processes may represent a useful chemopreventive, and even chemotherapeutic, strategy.

Bitter phytotastants showed a higher probability to exert both AIA and ACA, while sweet ones had a negative correlation with both AIA and ACA. This result suggests a potential beneficial implication of bitter taste receptor agonists and sweet taste receptor antagonists in slowing down the progression of inflammation to cancer. TAS2Rs, for example, are seen as promising targets in treating inflammation [14,60] and as potential targets in cancer prevention and treatment [16]. Among chemical classes, only flavonoids, which are most frequently bitter, had a positive association with both anti-inflammatory and anti-cancer activities, confirming the predictive primacy of taste over chemical classes. Experimental studies and clinical trials are necessary to confirm these results in the future.

## Figures and Tables

**Table 1:** The correlation between anti-inflammatory/anti-cancer activity and taste. Legend: PDA = pharmacodynamic activity, AIA—anti-inflammatory activity, ACA—anti-cancer activity. Note: The third column contains the number of phytocompounds in each of the 4 cells of the 2 × 2 tables used for performing Fisher’s exact test: a = taste-yes, AIA/ACA-yes; b = taste-yes, AIA/ACA-no; c = taste-no, AIA/ACA-yes; d = taste-no, AIA/ACA-no. As multiple comparisons were performed, the significance level (commonly set at 0.05) was lowered according to Bonferroni correction: the corrected significance level was 0.0035 (i.e., 0.05 divided by 14, the number of comparisons). Statistically significant results are in bold type.

Taste/Orosensation	PDA	(a, b, c, d)	Odds Ratio (OR)	95% Confidence Interval for OR	*p*-Value
Astringent	ACA	(75, 17, 386, 146)	1.67	0.97-2.99	0.073
Bitter (flavonoids only)	ACA	(57, 0, 5, 2)	Infinity	__	0.01
**Bitter**	**ACA**	**(324, 90, 137, 73)**	**1.92**	**1.32–2.77**	**7 × 10^−4^**
Pungent	ACA	(64, 18, 397, 145)	1.3	0.75–2.32	0.42
Salty	ACA	(2, 2, 459, 161)	0.35	0.04–3.4	0.28
Sour	ACA	(29, 19, 432, 144)	0.51	0.28–0.95	0.039
**Sweet**	**ACA**	**(55, 45, 406, 118)**	**0.36**	**0.23–0.56**	**1 × 10^−5^**
Umami	ACA	(4, 5, 457, 158)	0.28	0.07–1.11	0.057
Astringent	AIA	(82, 10, 429, 103)	1.97	1.01–4.13	0.056
**Bitter**	**AIA**	**(353, 61, 158, 52)**	**1.9**	**1.25–2.88**	**0.003**
Pungent	AIA	(70, 12, 441, 101)	1.34	0.71–2.66	0.44
Salty	AIA	(3, 1, 508, 112)	0.66	0.07–17.56	0.55
Sour	AIA	(37, 11, 474, 102)	0.72	0.36–1.53	0.43
Sweet	AIA	(73, 27, 438, 86)	0.53	0.32–0.88	0.016
Umami	AIA	(6, 3, 505, 110)	0.44	0.11–2.16	0.21

**Table 2:** The correlation between anti-cancer/anti-inflammatory activity and chemical classes. Legend: ACA—anti-cancer activity, AIA—anti-inflammatory activity, chemClass—chemical class. Note: The third column contains the number of phytocompounds in each of the 4 cells of the 2 × 2 tables built on the model mentioned in Section 4 (used for performing Fisher’s exact test): a = chemClass-yes, AIA/ACA-yes; b = chemClass-yes, AIA/ACA-no; c = chemClass-no, AIA/ACA-yes; d = chemClass-no, AIA/ACA-no. As multiple comparisons were performed, the significance level (commonly set at 0.05) was lowered according to Bonferroni correction: the corrected significance level was 0.001 (i.e., 0.05 divided by 42, the number of comparisons). Statistically significant results are in bold type.

Chemical Class	PDA	(a, b, c, d)	Odds Ratio (OR)	95% Confidence Interval for OR	*p*-Value
alkaloids	ACA	(71, 37, 390, 126)	0.62	0.4–0.98	0.04
**flavonoids**	**ACA**	**(62, 2, 399, 161)**	**12.48**	**3.58–76.76**	**7 × 10^−7^**
flavonoid glycosides	ACA	(33, 4, 428, 159)	3.06	1.15–10.28	0.032
**saccharides**	**ACA**	**(14, 18, 447, 145)**	**0.25**	**0.12–0.52**	**0.0002**
**amino acids**	**ACA**	**(9, 21, 452, 142)**	**0.14**	**0.06–0.3**	**3 × 10^−7^**
monoterpenoids	ACA	(21, 6, 440, 157)	1.25	0.51–3.44	0.82
triterpenoids	ACA	(20, 2, 441, 161)	3.65	0.97–23.34	0.082
coumarins	ACA	(20, 1, 441, 162)	7.33	1.33–155.28	0.022
sesquiterpenoids	ACA	(18, 3, 443, 160)	2.16	0.68–9.33	0.31
phenolic others	ACA	(16, 3, 445, 160)	1.92	0.6–8.33	0.43
phenolic acids	ACA	(14, 4, 447, 159)	1.24	0.42–4.45	1
short aliphatic acids	ACA	(9, 9, 452, 154)	0.34	0.13–0.9	0.028
tannins	ACA	(14, 1, 447, 162)	5.07	0.89–109.2	0.13
diterpenoids	ACA	(12, 2, 449, 161)	2.15	0.54–14.29	0.54
sulfur compounds	ACA	(8, 6, 453, 157)	0.46	0.15–1.45	0.21
monoterpenoid glycosides	ACA	(12, 1, 449, 162)	4.32	0.74–94.12	0.2
steroid glycosides	ACA	(10, 1, 451, 162)	3.59	0.59–79.2	0.3
fatty acids	ACA	(8, 2, 453, 161)	1.42	0.32–9.89	1
phenolic acid esters	ACA	(7, 3, 454, 160)	0.82	0.21–3.96	0.73
phenylpropanoids (others) *	ACA	(7, 3, 454, 160)	0.82	0.21–3.96	0.73
triterpenoid glycosides	ACA	(9, 1, 452, 162)	3.22	0.52–71.78	0.47
**alkaloids**	**AIA**	**(74, 34, 437, 79)**	**0.39**	**0.25–0.64**	**0.0002**
**flavonoids**	**AIA**	**(63, 1, 448, 112)**	**15.72**	**3.03–324.24**	**5 × 10^−5^**
flavonoid glycosides	AIA	(34, 3, 477, 110)	2.61	0.87–10.89	0.12
**saccharides**	**AIA**	**(16, 16, 495, 97)**	**0.2**	**0.09–0.41**	**2 × 10^−5^**
amino acids	AIA	(25, 5, 486, 108)	1.11	0.44–3.33	1
monoterpenoids	AIA	(26, 1, 485, 112)	5.99	1.11–125.95	0.043
triterpenoids	AIA	(19, 3, 492, 110)	1.42	0.45–6.1	0.78
coumarins	AIA	(20, 1, 491, 112)	4.56	0.83–96.63	0.15
sesquiterpenoids	AIA	(18, 3, 493, 110)	1.34	0.42–5.79	0.78
phenolic others	AIA	(16, 3, 495, 110)	1.18	0.37–5.17	1
phenolic acids	AIA	(16, 2, 495, 111)	1.79	0.46–11.67	0.75
short aliphatic acids	AIA	(11, 7, 500, 106)	0.33	0.13–0.93	0.029
tannins	AIA	(14, 1, 497, 112)	3.15	0.55–68.05	0.33
diterpenoids	AIA	(13, 1, 498, 112)	2.92	0.5–63.36	0.48
sulfur compounds	AIA	(10, 4, 501, 109)	0.54	0.17–2.04	0.3
monoterpenoid glycosides	AIA	(12, 1, 499, 112)	2.69	0.46–58.69	0.48
steroid glycosides	AIA	(8, 3, 503, 110)	0.58	0.16–2.76	0.43
fatty acids	AIA	(7, 3, 504, 110)	0.51	0.13–2.46	0.4
phenolic acid esters	AIA	(9, 1, 502, 112)	2.01	0.32–44.8	0.7
phenylpropanoids (others) *	AIA	(8, 2, 503, 111)	0.88	0.2–6.16	1
triterpenoid glycosides	AIA	(10, 0, 501, 113)	Infinity	__	0.22

Legend.* Other than the main classes of (iso)flavonoids, e.g., coumarins, stilbenes, lignans, and hydroxycinnamic acids, hydroxycinnamic acids derivatives (such as amides or esters), cinnamic acid esters, methoxyphenols.

**Table 3:** The correlation between anti-cancer/anti-inflammatory activity and pooled chemical classes. Legend: ACA—anti-cancer activity, AIA—anti-inflammatory activity, chemClass—pooled chemical class. Note: The third column contains the number of phytocompounds in each of the 4 cells of the 2 × 2 tables built on the model mentioned in Section 4 (used for performing Fisher’s exact test): a = chemClass-yes, AIA/ACA-yes; b = chemClass-yes, AIA/ACA-no; c = chemClass-no, AIA/ACA-yes; d = chemClass-no, AIA/ACA-no. As multiple comparisons were performed, the significance level (commonly set at 0.05) was lowered according to Bonferroni correction: the corrected significance level was 0.0015 (i.e., 0.05 divided by 34, the number of comparisons). Statistically significant results are in bold type.

Pooled Chemical Class	PDA	(a, b, c, d)	Odds Ratio (OR)	95% Confidence Interval for OR	*p*-Value
**alkaloids**	**AIA**	**(76, 34, 435, 79)**	**0.41**	**0.25–0.66**	**0.0003**
amino acids	AIA	(25, 5, 486, 108)	1.11	0.44–3.33	1
coumarins	AIA	(22, 1, 489, 112)	5.03	0.92–106.32	0.01
diterpenoids	AIA	(16, 1, 495, 112)	3.62	0.64–77.5	0.33
fatty compounds	AIA	(16, 6, 495, 107)	0.58	0.23–1.64	0.26
**flavonoids**	**AIA**	**(97, 4, 414, 109)**	**6.37**	**2.5–20.81**	**9 × 10^−6^**
monoterpenoids	AIA	(48, 3, 463, 110)	3.8	1.29–15.65	0.014
phenolic acids	AIA	(27, 3, 484, 110)	2.04	0.67–8.62	0.33
phenolic others	AIA	(19, 3, 492, 110)	1.42	0.45–6.1	0.78
phenylpropanoids	AIA	(9, 2, 502, 111)	1	0.23–6.84	1
**saccharides**	**AIA**	**(16, 16, 495, 97)**	**0.2**	**0.09–0.41**	**2 × 10^−5^**
sesquiterpenoids	AIA	(18, 5, 493, 108)	0.79	0.3–2.43	0.59
short aliphatic acids	AIA	(11, 7, 500, 106)	0.33	0.13–0.93	0.029
steroids	AIA	(13, 3, 498, 110)	0.96	0.29–4.26	1
sulfur compounds	AIA	(10, 5, 501, 108)	0.43	0.15–1.42	0.16
tannins	AIA	(14, 1, 497, 112)	3.15	0.55–68.05	0.33
triterpenoids	AIA	(29, 3, 482, 110)	2.2	0.73–9.26	0.24
alkaloids	ACA	(73, 37, 388, 126)	0.64	0.41–1.01	0.056
**amino acids**	**ACA**	**(9, 21, 452, 142)**	**0.14**	**0.06–0.3**	**3 × 10^−7^**
coumarins	ACA	(21, 2, 440, 161)	3.84	1.03–24.49	0.055
diterpenoids	ACA	(13, 4, 448, 159)	1.15	0.39–4.16	1
fatty compounds	ACA	(15, 7, 446, 156)	0.75	0.3–2	0.62
**flavonoids**	**ACA**	**(95, 6, 366, 157)**	**6.78**	**3.08–17.41**	**5 × 10^−8^**
monoterpenoids	ACA	(40, 11, 421, 152)	1.31	0.67–2.73	0.51
phenolic acids	ACA	(23, 7, 438, 156)	1.17	0.51–2.99	0.83
phenolic others	ACA	(19, 3, 442, 160)	2.29	0.73–9.83	0.22
phenylpropanoids	ACA	(8, 3, 453, 160)	0.94	0.25–4.44	1
**saccharides**	**ACA**	**(14, 18, 447, 145)**	**0.25**	**0.12–0.52**	**0.0002**
sesquiterpenoids	ACA	(18, 5, 443, 158)	1.28	0.49–3.93	0.81
short aliphatic acids	ACA	(9, 9, 452, 154)	0.34	0.13–0.9	0.028
steroids	ACA	(15, 1, 446, 162)	5.44	0.96–116.79	0.083
sulfur compounds	ACA	(8, 7, 453, 156)	0.39	0.14–1.16	0.078
tannins	ACA	(14, 1, 447, 162)	5.07	0.89–109.2	0.13
triterpenoids	ACA	(29, 3, 432, 160)	3.58	1.19–14.97	0.024

**Table 4:** The correlation between anti-inflammatory (AIA) and anti-cancer activity (ACA) for all tastes and for each taste is taken separately. Salty and umami are absent due to the low count of corresponding phytocompounds. Note: The second column contains the number of phytocompounds in each of the 4 cells of the 2 × 2 tables built on the model mentioned in Section 4 (used for performing Fisher’s exact test):a = AIA-no, ACA-no; b = AIA-yes, ACA-no; c = AIA-no, ACA-yes; d = AIA-yes, ACA-yes. As multiple comparisons were performed, the significance level (commonly set at 0.05) was lowered according to Bonferroni correction: the corrected significance level was 0.01 (i.e., 0.05 divided by 6, the number of comparisons).

Taste	(a, b, c, d)	Odds Ratio (OR)	95% Confidence Interval for OR	*p*-Value
All tastes	(81, 32, 82, 429)	13.16	8.25–21.32	3 × 10^−30^
Astringent	(9, 1, 8, 74)	75.56	10.69–1847.1	3 × 10^−7^
Bitter	(40, 21, 50, 303)	11.44	6.27–21.31	4 × 10^−16^
Pungent	(8, 4, 10, 60)	11.45	2.93–51.13	0.0004
Sour	(10, 1, 9, 28)	28.62	4.05–700.41	0.0001
Sweet	(22, 5, 23, 50)	9.33	3.26–30.75	1 × 10^−5^

**Table 5:** The correlation between anti-inflammatory (AIA) and anti-cancer activity (ACA) for each chemical class is taken separately. Note: The second column contains the number of phytocompounds in each of the 4 cells of the 2 × 2 tables built on the model mentioned in Section 4 (used for performing Fisher’s exact test): a = AIA-no, ACA-no; b = AIA-yes, ACA-no; c = AIA-no, ACA-yes; d = AIA-yes, ACA-yes. Inf = infinity. As multiple comparisons were performed, the significance level (commonly set at 0.05) was lowered according to Bonferroni correction: the corrected significance level was 0.002 (i.e., 0.05 divided by 21, the number of comparisons). Statistically significant results are in bold type.

Chemical Class	(a, b, c, d)	Odds Ratio (OR)	95% Confidence Interval for OR	*p*-Value
**alkaloids**	**(24, 13, 10, 61)**	**10.93**	**3.97–32.64**	**2 × 10^−7^**
flavonoids	(1, 1, 0, 62)	Inf	0.79–Inf	0.031
flavonoid glycosides	(2, 2, 1, 32)	25.21	0.98–1904.49	0.026
saccharides	(12, 6, 4, 10)	4.73	0.89–30.52	0.073
amino acids	(5, 16, 0, 9)	Inf	0.41-Inf	0.29
monoterpenoids	(1, 5, 0, 21)	Inf	0.09-Inf	0.22
triterpenoids	(1, 1, 2, 18)	7.55	0.08–738.06	0.26
coumarins	(0, 1, 1, 19)	0	0–770.62	1
sesquiterpenoids	(2, 1, 1, 17)	23.02	0.74–2065.5	0.041
phenolic others	(2, 1, 1, 15)	20.38	0.65–1840.77	0.051
phenolic acids	(2, 2, 0, 14)	Inf	0.75-Inf	0.039
short aliphatic acids	(6, 3, 1, 8)	13.25	1–819.63	0.05
tannins	(1, 0, 0, 14)	Inf	0.36-Inf	0.067
diterpenoids	(0, 2, 1, 11)	0	0–233.15	1
sulfur compounds	(3, 3, 1, 7)	5.99	0.33–417.16	0.24
monoterpenoid glycosides	(0, 1, 1, 11)	0	0–464.61	1
steroid glycosides	(1, 0, 2, 8)	Inf	0.07-Inf	0.27
fatty acids	(1, 1, 2, 6)	2.65	0.03–273.2	1
phenolic acid esters	(1, 2, 0, 7)	Inf	0.06-Inf	0.3
phenylpropanoids	(1, 2, 1, 6)	2.65	0.03–273.2	1
triterpenoid glycosides	(0, 1, 0, 9)	0	0-Inf	1

**Table 6:** The 2 × 2 contingency table used for performing Fisher’s exact test (AIA/ACA = anti-inflammatory/anti-cancer activity).

	No AIA/ACA	AIA/ACA
No Taste/Chemical class	a	b
Taste/Chemical class	c	d

## Data Availability

The data used in this study are freely available in PMTDB (http://plantmoleculartastedb.org, accessed on 1 February 2023), PubMed, ScienceDirect, Google Scholar.

## Abbreviations

ACA	anti-cancer activity
AIA	anti-inflammatory activity
chemClass	chemical class
PMTDB	PlantMolecularTasteDB
PDA	pharmacodynamic activity
TASRs	taste receptors
